# Immunomodulatory Effects of Rhinovirus and Enterovirus Infections During the First Year of Life

**DOI:** 10.3389/fimmu.2020.567046

**Published:** 2021-02-11

**Authors:** Terhi Ruohtula, Anita Kondrashova, Jussi Lehtonen, Sami Oikarinen, Anu-Maaria Hämäläinen, Onni Niemelä, Aleksandr Peet, Vallo Tillmann, Janne K. Nieminen, Jorma Ilonen, Mikael Knip, Outi Vaarala, Heikki Hyöty, the DIABIMMUNE Study Group, Mikael Knip

**Affiliations:** ^1^ Clinicum, University of Helsinki, Helsinki, Finland; ^2^ Faculty of Medicine and Health Technology, Tampere University, Tampere, Finland; ^3^ Department of Pediatrics, Jorvi Hospital, Helsinki University Hospital, Espoo, Finland; ^4^ Department of Laboratory Medicine and Medical Research Unit, Seinäjoki Central Hospital and University of Tampere, Seinäjoki, Finland; ^5^ Department of Pediatrics, University of Tartu and Tartu University Hospital, Tartu, Estonia; ^6^ Pediatric Research Center, Children’s Hospital, University of Helsinki and Helsinki University Hospital, Helsinki, Finland; ^7^ Immunogenetics Laboratory, University of Turku, Turku, Finland; ^8^ Research Program for Clinical and Molecular Metabolism, Faculty of Medicine, University of Helsinki, Helsinki, Finland; ^9^ Folkhälsan Research Center, Helsinki, Finland; ^10^ Tampere Center for Child Health Research, Tampere University Hospital, Tampere, Finland; ^11^ Fimlab Laboratories, Pirkanmaa Hospital District, Tampere, Finland

**Keywords:** enterovirus, rhinovirus, regulatory T cell, cytokine, type 1 diabetes

## Abstract

Early childhood infections have been implicated in the development of immune-mediated diseases, such as allergies, asthma, and type 1 diabetes. We set out to investigate the immunomodulatory effects of early viral infections experienced before the age of one year on the peripheral regulatory T cell population (Treg) and circulating cytokines in a birth-cohort study of Estonian and Finnish infants. We show here a temporal association of virus infection with the expression of FOXP3 in regulatory T cells. Infants with rhinovirus infection during the preceding 30 days had a higher FOXP3 expression in Treg cells and decreased levels of several cytokines related to Th1 and Th2 responses in comparison to the children without infections. In contrast, FOXP3 expression was significantly decreased in highly activated (CD4+CD127−/loCD25+FOXP3high) regulatory T cells (TregFOXP3high) in the infants who had enterovirus infection during the preceding 30 or 60 days. After enterovirus infections, the cytokine profile showed an upregulation of Th1- and Th17-related cytokines and a decreased activation of CCL22, which is a chemokine derived from dendritic cells and associated with Th2 deviation. Our results reveal that immunoregulatory mechanisms are up-regulated after rhinovirus infections, while enterovirus infections are associated with activation of proinflammatory pathways and decreased immune regulation.

## Introduction

The immune system maturates rapidly during the first year of life. The immunological pathways that mediate the pathogenesis of immune-mediated diseases are also often programmed during this period. Early infections can influence this maturation process and either increase or decrease the risk of immune-mediated diseases. Among the most widely studied questions is the possible role of enterovirus infections (including rhinoviruses) in the development of asthma and type 1 diabetes (T1D), but the exact mechanisms of these associations are not fully understood (1). One of the most feasible hypothesis is that early enterovirus infections influence the regulatory elements of the immune system.

FOXP3-expressing regulatory T cells (Tregs) play crucial roles in maintaining tolerance to self-antigens and in regulating excessive inflammation in infectious diseases. They comprise 1%–10% of thymic and peripheral CD4+ T cells. It is well established that microbes can induce Tregs (iTregs), which may play a key role in the regulation of harmful immune responses. However, iTreg responses are dynamic, and the factors that determine their nature are complex and are still being elucidated. Viral infections have potent effects on cytokine production, influence T cell differentiation, and induce a broad range of iTreg subsets. Virus-induced Tregs are generally antigen-specific, inhibit proliferation and cytokine production of CD4+ and CD8+ T cells, and affect the maturation of dendritic cells, activation of natural killer cells, and immunoglobulin production of B cells (2, 3). iTreg cells inhibit T cell responses either indirectly through the production of regulatory cytokines such as TGF-β or IL-10 or directly through cell-to-cell contact (4, 5). iTregs form a memory pool after the resolution of the infection and these memory iTregs can rapidly be activated to suppress the collateral tissue damage and inflammation caused by recall activation of effector T cells in the context of re-infection (6). In addition to CD4+ regulatory T cell subsets, also CD8+ T cells have been described to inhibit T cell proliferation and cytokine production (7). The regulatory effects of CD8+ T cells have mainly been associated with chronic, persistent infectious diseases such as leprosy, HIV, Epstein-Barr virus, hepatitis C, and tuberculosis (8–12).

Accumulating evidence shows that bacterial microbiota may regulate the activation of iTregs, but less is known about the possible effects of viral infections in young children. To shed light on this important question, the current study investigates the immunomodulatory effects of early viral infections experienced before the age of one year on the peripheral Treg cell population and circulating cytokines.

## Materials and Methods

### Study Subjects

The study cohort included altogether 136 children from Estonia (EST, N = 71; 36 male) and Finland (FIN, N = 65; 36 male) who were prospectively followed from birth. Blood samples were collected for the flow cytometry analyses of regulatory T cells from 136 infants (71 EST) at the age of 3 months (111 children; 56 EST), 6 months (45 children; 18 EST), and 12 months of age (100 children; 43 EST). Serum samples for cytokine analysis were collected from 136 children (75 EST) at the same time points as Treg samples (103/56; 100/51; and 101/45 samples/EST, respectively). Stool samples for virus analyses were collected every month during the first year of life starting at the age of 1 month [altogether 1,063 samples from 116 children, 54 EST, (29/34 male, EST/FIN, respectively), on an average eight samples/child]. Stool samples were frozen immediately after sample collection at home at −20**°**C and transported to the study center frozen as soon as possible, where the samples were stored at −80°C until analyzed.

Children were recruited during the years 2009–2010 in Estonia and Finland for the DIABIMMUNE (Pathogenesis of T1D: testing the hygiene hypothesis) study (13). All children carried T1D associated HLA risk genotypes (HLA-DQA1*05-DQB1*02/*0302, *0302/x genotypes [x≠*02, *0301 or *0602] or HLA-DQA1*05-DQB1*02/x genotypes). Local ethics committees approved the study protocols, and the study was carried out in accordance with the Declaration of Helsinki. The parents of the infants gave their informed written consent to the study.

### PCR Analyses of Stool Samples From Children

RT-PCR was used for screening of stool samples (N = 1,063) for enterovirus, rotavirus, norovirus, parechovirus, and rhinovirus, as previously described. First, a 10% stool suspension was prepared from the original stool sample, and viral RNA was extracted using the modified Qiagen RNeasy96 kit (QIAGEN, Germany). Viral RNA from stool samples was analyzed with previously described PCR methods (14–18). The primers and probes and the concentration of the oligonucleotides in the qPCR reactions are listed in **Supplementary Table 1**.

### Cytokine/Chemokine Analysis of Serum Samples

Unthawed serum samples were used for the cytokine/chemokine analysis, as repeated freezing and thawing of the samples decreases the concentrations of detected analytes. Cytokines/chemokines detected were: EGF, Eotaxin, FGF-2, Flt-3L, Fractalkine, G-CSF, GM-CSF, GRO, IFNα2, IFNγ, IL-1RA, IL-1α, IL-1β, IL-2, IL-3, IL-4, IL-5, IL-6, IL-7, IL-8, IL-9, IL-10, IL-12P40, IL-12P70, IL-13, IL-15, IL-17A, IP-10 (CXCL10), MCP-1, MCP-3, MDC (CCL22), MIP-1α, MIP-1β (CCL4), TGF-α, TNFα, TNFβ, sCD40L, and VEGF. Cytokine concentrations were assessed as pg/ml using multiplex ELISA (MILLIPLEX MAP Human Cytokine/Chemokine Magnetic bead 38-plex Panel, Millipore, Billerica, MA) according to the manufacturer’s instructions, except for adding a third wash with washing buffer to the plates and replacing sheath fluid with phosphate-buffered saline (PBS) to the samples for Luminex reading. The Bio-Rad Bio-Plex 200 System (Bio-Rad Laboratories, Hercules, CA) instrument was used with the Bio-Plex Manager 5.0 program to run plates and generate quantitative data.

### Analyses of Regulatory T Cells

We used Flow cytometry for samples obtained at the age of 3, 6, and 12 months. For flow cytometry, 200 µl of fresh heparinized blood was added to monoclonal antibodies for 20 min, and erythrocytes were then lysed with BD-FACS Lysing Solution. After two washes with washing buffer consisting of 5% fetal bovine serum and 0.02% (w/v) sodium azide in phosphate-buffered saline, cells were suspended in 1% (w/v) paraformaldehyde in PBS and stored overnight at 4°C. At least 1 × 10^6^ events were acquired from each sample on a FACSCalibur™ and analyzed with the FlowJo™ software. The samples were compensated post-acquisition with FlowJo™ software. To assess the number of circulating CD4+CD25highFOXP3+ T cells in the samples, we gated first CD4+ cells, and then the CD25+CD127−/lo population. The expression of FOXP3 protein was analyzed in these cell populations. CD4+CD25highFOXP3+ expression was quantified as median fluorescence intensity (MFI) in arbitrary units (AU) after subtraction of the negative-control antibody intensity. Intensity values over the 97.5 percentile of the negative-control antibody were regarded as positive. Intensities were calibrated to a set of particles containing known amounts of fluorescein isothiocyanate (see gating in **Supplementary Figure 1A**).

All products used in this study are listed in **Supplementary Table 2**.

### Statistical Methods

The association of virus infections with Tregs and serum cytokines were analyzed separately for respiratory infections (rhinovirus) and enteral infections (norovirus, rotavirus, parechovirus, and enterovirus combined). The analyses were performed by categorizing children into virus-positive and -negative groups and by comparing Treg activity between these two groups. In addition, time-dependent effects were evaluated by analyzing infections in different time-windows in relation to the collection of the Treg sample, including infections within 14 days, 30 days, and 60 days before Treg analyses. Wilcoxon rank-sum test (R-version 3.6.2) was used for statistical analysis of the data. A *p* < 0.05 was considered statistically significant. The *p* values of each independent analysis were corrected for the number of multiple comparisons (N = 5 in the analysis of virus-Treg associations; N = 30 in the analysis of virus-cytokine/chemokine associations, since a majority of the samples had undetectable concentrations of eight cytokines and these cytokines were excluded from the statistical analysis).

## Results

### Viruses Detected in Stool Samples During the First Year of Life

**Figure 1:**
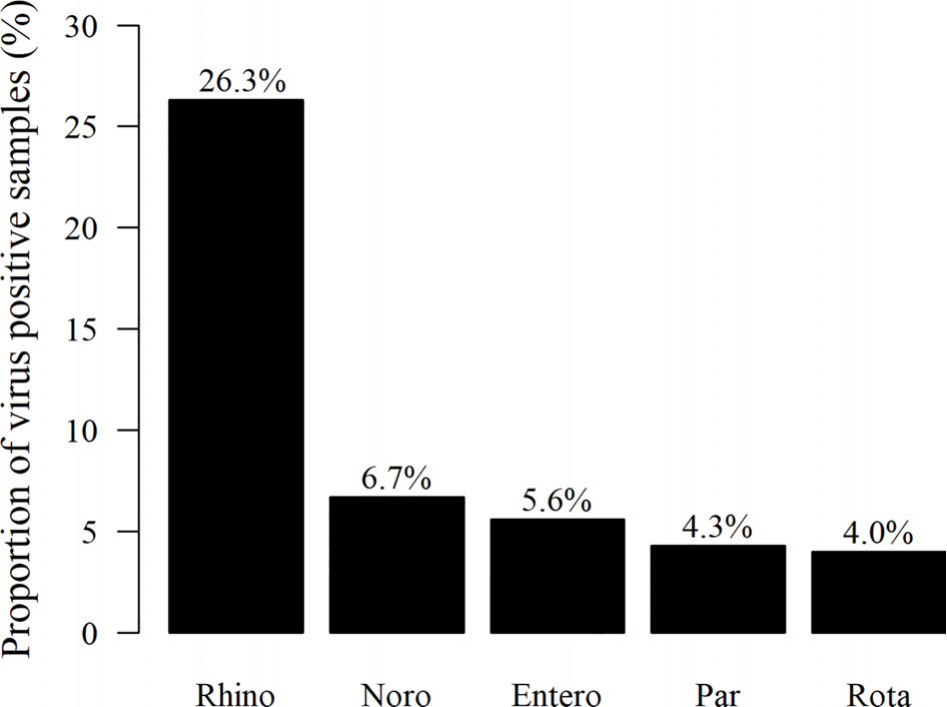
Proportion (%) of stool samples positive for either enteral or respiratory viruses (433 samples) tested, from all 1,063 stool samples collected during the first 12 months of life. The most frequently detected virus was rhinovirus (Rhino) in 280 of the virus-positive samples, followed by norovirus G1/G2 (Noro) (71), enterovirus (Entero) (59), parechovirus (Par) (46), and rotavirus (Rota) (42 samples), respectively.

**Figure 2:**
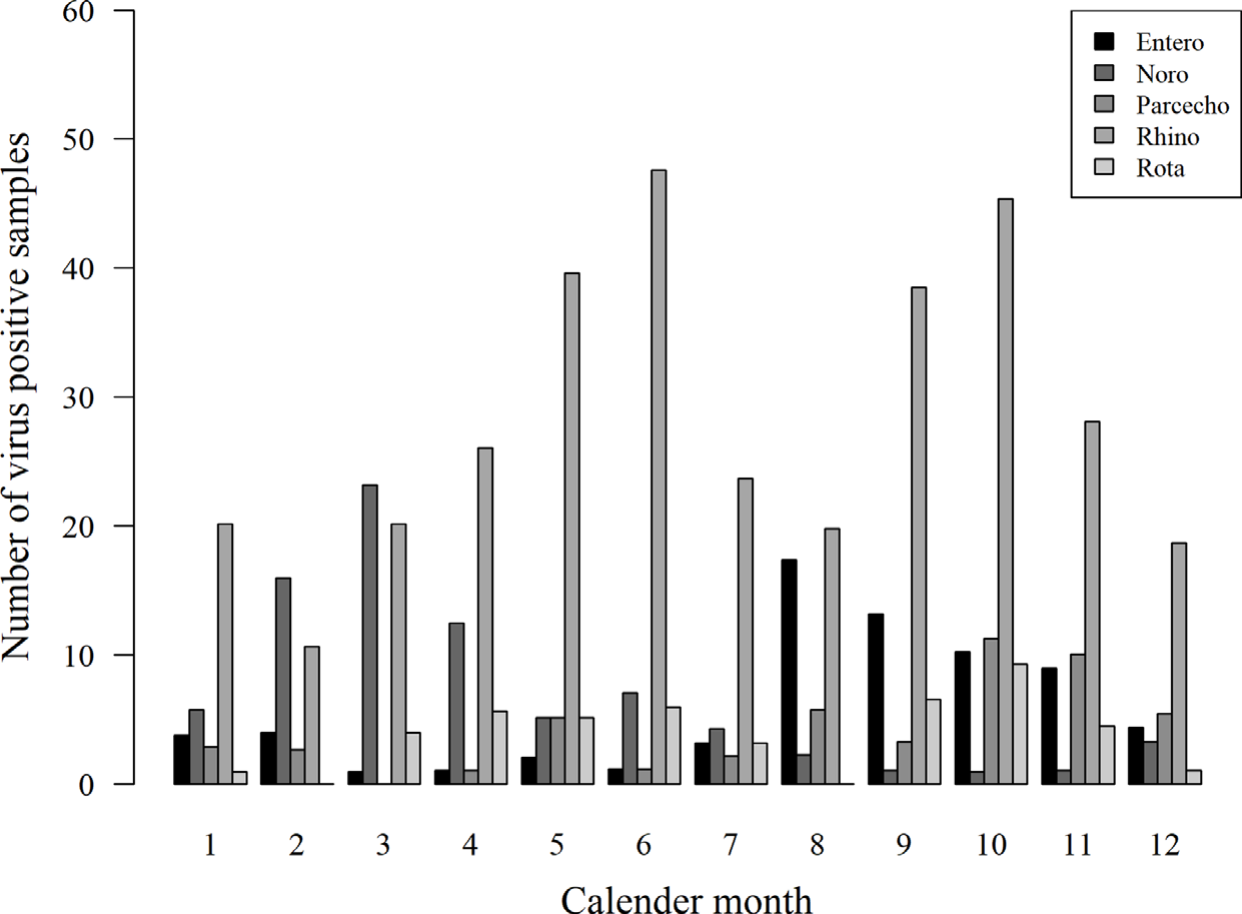
Seasonality of virus detection in stool samples. Enterovirus (Entero), parechovirus (Parecho), and rotavirus (Rota) were most frequently detected during autumn months, while norovirus (Noro) was most frequent from February to March. Rhinovirus (Rhino) was most frequent in May-June and September-October.

The presence of viruses was analyzed from all stool samples collected during the first 12 months of life. Of the altogether 1,063 samples, 433 (40.7%) were positive for at least one of the tested viruses. Among the 116 children, 108 (93.1%) were virus-positive at least once, and eight (6.9%) were virus-negative in all samples. The most frequently detected virus was rhinovirus in 280 of the samples (26.3%), followed by norovirus G1 and G2 (71 samples/6.7%), enterovirus (59/5.6%), parechovirus (46/4.3%), and rotavirus (42/4.0%; **Figure 1**). Rhinoviruses were most frequently detected during the first 6 months of life, while positivity for enteroviruses, noroviruses, and parechoviruses was highest at the age of 6–12 months (**Table 1**). Rotavirus was most frequently seen at the age of 2 and 3 months in Finland, while there was only one rotavirus positive sample in Estonia. This is likely due to the introduction of a live attenuated rotavirus vaccine in Finland at the age of 2, 3, and 5 months while rotavirus vaccination was not used in Estonia during the study. Enterovirus, parechovirus, and rotavirus were most frequently detected during autumn months, while norovirus was most frequent from February to March. Rhinovirus was most frequent in May-June and September-October (**Figure 2**).

**Table 1:** Summary of virus positivity in stool samples at different ages.

Age month	No of samples	Rhino	Entero	Noro	Parecho	Rota	Total
1	46	13 (28.3)	0 (0.0	1 (2.2)	0 (0.0)	0 (0.0)	14 (30.4)
2	92	32 (34.8)	0 (0.0)	3 (3.3)	2 (2.2)	14 (15.2)	51 (55.4)
3	106	37 (34.9)	2 (1.9)	3 (2.8)	2 (1.9)	17 (16)	61 (57.5)
4	99	41 (41.4)	0 (0.0)	3 (3.0	1 (1.0)	5 (5.1)	50 (50.5)
5	100	34 (34.0)	3 (3.0)	4 (4.0)	2 (2.0)	3 (3.0)	46 (46.0)
6	104	37 (35.6)	8 (7.7)	7 (6.7)	7 (6.7)	1 (1.0)	60 (57.7)
7	86	17 (19.8)	6 (7.0)	7 (8.1)	5 (5.8)	0 (0.0)	35 (40.7)
8	87	13 (14.9)	11 (12.6)	7 (8.0)	(11.5)	0 (0.0)	41 (47.1)
9	88	20 (22.7)	6 (6.8)	7 (8.0)	4 (4.5)	1 (1.1)	38 (43.2)
10	85	11 (12.9)	8 (9.4)	5 (5.9)	5 (5.9)	1 (1.2)	30 (35.3)
11	84	12 (14.3)	8 (9.5)	10 (11.9)	3 (3.6)	0 (0.0)	33 (39.3)
12	86	13 (15.1)	7 (8.1)	14 (16.3)	5 (5.8)	0 (0.0)	39 (45.3)

### FOXP3 Expression in Treg Cells Decreases and Stays Lower Up to 60 Days After Enterovirus Infection

**Figure 3:**
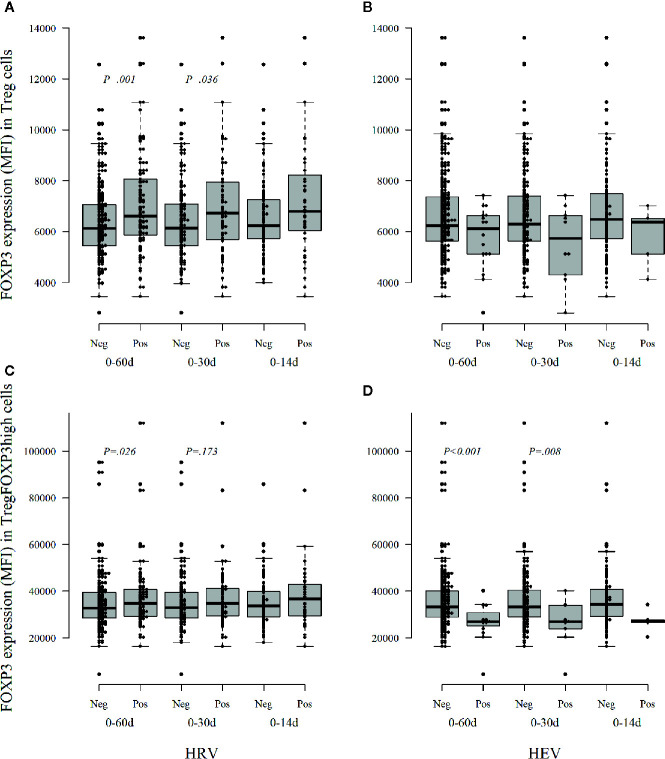
The intensity of FOXP3 expression, as median fluorescence intensity (MFI), in Treg cells **(A, B)** and activated Treg cells TregFOXP3high **(C, D)** during the first year of life according to the detection of viruses in stool samples within three different time windows, 14, 30, and 60 days, before the collection of a blood sample for Treg analyses. Neg, no viruses detected; Pos, at least one virus detected; HRV, human rhinovirus; HEV, human enterovirus. Medians are marked with a horizontal line, and whiskers show 5–95 percentiles. Correction for multiple comparisons (N = 5) retain statistical significance for *p* values ≤ 0.01.

To address the possible role of virus infections in the modulation of FOXP3 in Tregs, we analyzed the temporal association of virus infection with the expression of FOXP3 in Tregs. The children who had had at least one virus infection during the preceding 30 or 60 days showed increased expression of FOXP3 in Tregs in comparison to the children without virus infections in the same time period (*p* = 0.005 and *p* = 0.124). When the data were analyzed separately for various virus infections, the children with rhinovirus infection had higher FOXP3 expression in Treg cells than children without rhinovirus infection [p = 0.036 for the 30-day, and *p* = 0.001 for the 60-day period (*p* = 0.18 and *p* = 0.005 after correction for multiple comparisons); **Figure 3A**]. In contrast, the infants with enterovirus positivity within 30 days before Treg analyses showed a clear but statistically non-significant decrease in FOXP3 intensity in the total Treg population (**Figure 3B**).

In the analyses of the highly activated Treg cells (CD4+CD127−/loCD25+FOXP3high Tregs), the FOXP3 expression level was decreased in the infants who had enterovirus infection during the preceding 30 or 60 days period [*p* = 0.008 and *p* < 0.001 (*p* = 0.04 and *p* < 0.005 after correction for multiple comparisons); **Figure 3D**]. Again, the infants with preceding rhinovirus infection showed some increased expression of FOXP3 in TregFOXP3high cells [*p* = 0.173 and *p* = 0.026 for 30 and 60 days (non-significant after correction for multiple comparisons), **Figure 3C**].

Our results suggest that rhinovirus and enterovirus infections modulate FOXP3 expression and Treg maturation differently during the first year of life: enterovirus decreases the FOXP3 expression while rhinovirus may increase FOXP3 expression. In the analyses of the other viruses, no such changes were seen (data not shown).

### Association Between Virus Infections and Serum Cytokines

**Figure 4:**
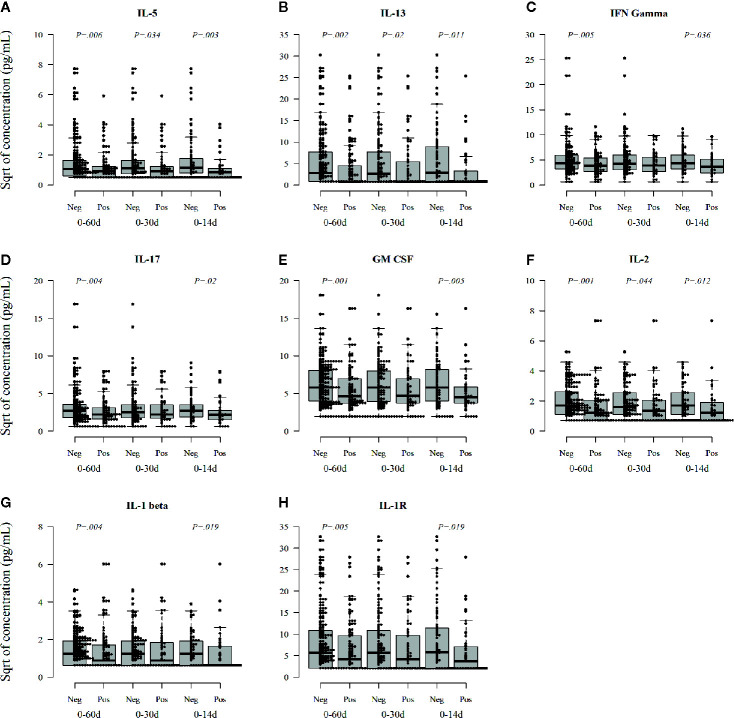
Rhinovirus infection-related changes in circulating cytokines (pg/ml) in serum in a window of 60, 30, and 14 days after infection. IL-5 **(A)**, IL-13 **(B)**, IFNγ **(C)**, Th17 **(D)**, GM-CSF **(E)**, IL-2 **(F)**, and Th1 cytokines IL-1β and sIL-1R **(G, H)**. Medians are marked with a horizontal line in the boxes, and whiskers show 5–95 percentiles. Correction for multiple comparisons (N = 30) retain statistical significance for *p* values ≤ 0.0016.

Next, we analyzed the levels of circulating cytokines, which could reflect the immunomodulatory effects of viral infections in the children. We found that the infants with preceding rhinovirus infection showed lower levels of several cytokines in comparison to those without preceding viral infections. The levels of T-helper 2 (Th2) phenotype related cytokines, IL-5 and IL-13 (**Figures 4A, B** **)**, as well as Th1 hallmark cytokine IFNγ and IL-2 (**Figures 4C, F** **)** and Th17 cytokine IL-17 (**Figure 4D** **)** and GM-CSF (**Figure 4E**), were significantly decreased after rhinovirus infection. Also, IL-1beta, and soluble IL-1R (**Figures 4G, H** **)**, which are related to the inflammasome activation were significantly reduced after rhinovirus infection. These associations were seen both when the cytokines were measured from samples taken soon (within 14 days) and long (within 60 days) after the infection.

**Figure 5:**
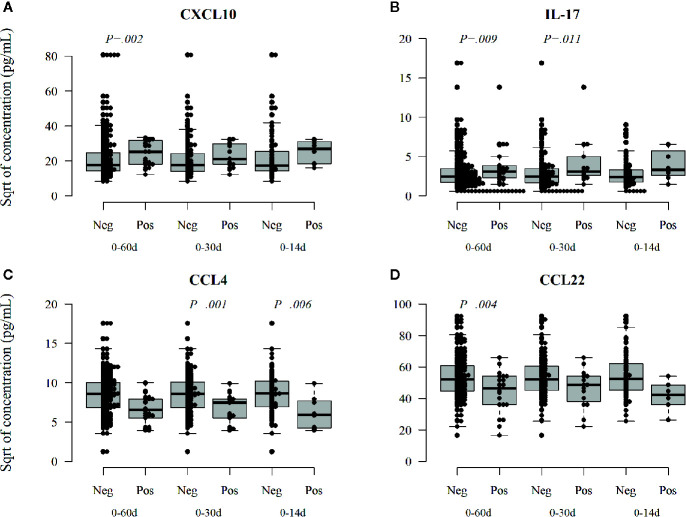
Enterovirus infection-related changes in circulating cytokines. Enterovirus infected infants had a different cytokine profile after the infection showing increased activation of Th1 and Th17, CXCL10 (IP-10) **(A)**, and Il-17 **(B)** cytokines but reduced activation of CCL4 (MIP-1β, **C**) and CCL22 (MDC, **D**). CCL22 is a chemokine derived from dendritic cells and associated with Th2 deviation. Medians are marked with a horizontal line, and whiskers show 5–95 percentiles. Correction for multiple comparisons (N = 30) retain statistical significance for *p* values ≤ 0.0016.

In contrast, in the infants with enterovirus infection increased levels of CXCL10 (IP-10, **Figure 5A** **)**, which is related to Th1 immunity and IL-17 (**Figure 5B** **)** were found after the infection, while CCL4 (MIP-1β) and CCL22 (MDC, **Figures 5C, D** **)**, remained decreased after enterovirus infection. Also, these associations were seen both in samples taken soon and long after the infection. IL-10 was not associated with rhinovirus or enterovirus infections but was increased after parechovirus infection (*p* = 0.009; *p* = 0.27 after correction for multiple comparisons). Norovirus infection was associated with a subsequent decrease in CXCL1 (GRO) (*p* = 0.006; *p* = 0.18 after correction), a chemoattractant for neutrophil infiltration. Rotavirus positivity was associated with a subsequently increased level of TNFalpha (*p* = 0.001; *p* = 0.03 after correction), data not shown.

## Discussion

In this prospective study, we set out to investigate the relationship between virus infections, the activation of iTreg cells, and circulating cytokines in young children. We focused on infections that occurred during the first year of life, as this period is critical for the maturation of the immune system. Viral infections are frequent during the first year of life, and their role as a risk factor for allergies, asthma, and autoimmune diseases, such as T1D, has gained attention during recent years (19–22). Treg cells are major regulators of immune homeostasis (23–26), but the possible short- and long-term effects of acute virus infections on the activation of iTreg cells are poorly understood.

According to the original hygiene hypothesis, infections during infancy could protect from allergies (27, 28), but the evidence supporting this has not been convincing (29). Actually, rhinoviruses and infection associated wheezing have been linked to the development of respiratory allergies or asthma later in life (25, 26, 29), particularly in infants with atopic immune deviation (30). On the other hand, we have recently observed that early exposure to rhinoviruses is inversely associated with later development of IgE sensitization, particularly in boys suggesting that rhinovirus infections may protect against IgE-mediated sensitization (31).

Here, we show that rhinovirus infections are associated with the up-regulation of FOXP3 expression in Treg cells and simultaneous down-regulation of a broad range of circulating cytokines, including Th1, Th2, and Th17 cytokines. These changes were seen in the children with rhinovirus positivity in the stool samples taken up to 60 days after virus positivity, suggesting that rhinovirus infections during the first year of life cause long-term immunomodulatory effects, which are characterized by an immunoregulatory phenotype. Our findings of rhinovirus-induced relative immunosuppression could explain the earlier observations of delayed induction of humoral immune response to rhinovirus, which may take 5 to 6 weeks, while protective antibodies are induced in 2 to 3 weeks after other viral infections. Many rhinoviruses use ICAM-1 as a receptor, and it has been speculated that this could result in the impaired induction of T cell responses (32). Our findings suggest that the up-regulation of regulatory mechanisms is pronounced after rhinovirus infection compared to other infections. This gives one possible mechanistic explanation for our finding of a protective association between early rhinovirus infections and later development of IgE-mediated sensitization (31), and this may also have implications in the persistence and spreading of rhinovirus infections in general.

Our findings do not have a direct link to the association of wheezing with rhinovirus infections, which is an acute event, while we here measured long-term immunomodulatory effects of rhinovirus infection. As the levels of circulating cytokines are known to increase with age during the first year of life (33) (see **Supplementary Figure 2** **)**, the down-regulation of circulating cytokines after rhinovirus infections could be interpreted as a possible delay in the maturation of the immune system. Further, *in vitro* studies to assess whether rhinoviruses deviate from other viruses would be pertinent. In atopic children with IgE sensitization, rhinovirus infection has been considered as a risk factor of later asthma. Earlier studies have shown that delayed maturation of the gut bacteriome and related immunological changes have been associated with the risk of atopic diseases, such as asthma (34, 35).

Our finding of the up-regulation of FOXP3 expression in Treg cells after rhinovirus infections is in line with studies showing activation of Treg cells in the context of virus infection (36–40). The activation of Treg cells in acute infection is associated with the resolution of viral immunopathology and thus can be considered beneficial, as demonstrated in animal models (6). In a recent study of H7N9 Influenza A infections in adults, a tendency to a decrease in Treg cells was seen during the disease progression and an increase during recovery (24). In regard to the risk of atopic diseases associated with Th2 deviation, it is of interest that Treg cells may acquire GATA-3 expression and show plasticity toward Th2 cells (41–43).

In contrast to rhinovirus infections, enterovirus infections were followed by a decreased FOXP3 expression in Tregs, particularly in the population of TregFOXP3high cells, which represent highly activated Tregs. The cytokine profile after enterovirus infections was also different, showing an upregulation of Th1 and Th17 responses, and decreased activation of CCL22, which is a chemokine derived from dendritic cells and associated with Th2 deviation. Up-regulation of Th1 and IL-17 and down-regulation of Th2 immunity after enterovirus infections could explain our earlier findings of an inverse association of certain enterovirus infections, namely, echo- and coxsackie-B-viruses, with low risk of IgE-mediated sensitization (28).

The present findings of the decreased FOXP3 expression in Tregs after enterovirus infections suggest impaired regulation of inflammation after enterovirus infections and are particularly interesting in the light of the enterovirus infections associated risk of complications, such as myocarditis, encephalitis, autonomous nervous dysregulation, and pulmonary edema. A low frequency of Treg cells was associated with the severity of EV71-associated pulmonary edema (44–46). Also, an increase in regulatory T cells alleviated Coxsackievirus B3 (CVB3) induced myocarditis in an animal model (47). Valproic acid, a histone deacetylase inhibitor that has anti-inflammatory effects, alleviated myocarditis in a mouse model by upregulating IL-10 in serum and heart tissues and promoting both the differentiation and suppressive function of Treg cells (48). Furthermore, the activation of Treg cells and M2 alternatively activated macrophages in the CVB3 H310A1 virus variant infection of C57Bl/6 mice protects them from myocarditis (49).

Enterovirus infections have also been linked to the initiation of islet autoimmunity and an increased risk of T1D (44–49). A recent report from the prospective TEDDY study, including the largest cohort of infants at genetic risk of T1D studied until now, found an association between prolonged enterovirus infections (long shedding of the virus into stools) and later appearance of islet autoantibodies (50). In the same cohort, enterovirus infection between age 1 and 2 years was associated with celiac disease-related autoimmunity (51). Also, in a recent Norwegian nested case-control study, enterovirus infection was associated with celiac disease (52).

Our findings of enterovirus infection associated down-regulation of FOXP3 in Treg cells combined with a deviation to an increased Th1 immunity provides evidence of a potential mechanism supporting the induction of autoimmune responses by an enterovirus. Among the different virus infections studied in our cohort, only enterovirus infection was associated with this kind of suppression of regulatory mechanisms. It is tempting to speculate that a lower expression of FOXP3 in Treg cells after enterovirus infections might lead to impaired suppression of anti-viral responses and tissue inflammation, which could contribute to the induction of autoimmunity. The mechanisms behind the immunomodulatory effects on the host immune system induced by enterovirus infection remains to be elucidated.

Due to the blood volume restrictions and the number of PBMCs available from these young infants, we could not study the memory Treg or memory Th17 cell populations. To our knowledge, there are no previous studies, which would have evaluated memory Tregs in enterovirus or rhinovirus infections. However, an earlier study has shown that children infected with enterovirus EV71 have higher frequencies of Th17 cells and serum IL-17 concentrations, suggesting that such cells are induced during the infections (53). In addition, IL-17 production has also been shown to be associated with a more severe course of enterovirus infection (54, 55). Altogether, the results of the present study emphasize the need for further studies on the Treg/IL-17 axis, including cells with the memory phenotype, to understand the long-term effects of enterovirus and rhinovirus infections on the regulation of the immune system in young infants.

One of the limitations of the present study is that due to ethical reasons, the Treg cells studied are from peripheral blood, while the detection of viruses was done in stool samples. Another limitation is that our viral analyses covered only certain viruses. Therefore, further studies are needed to get an overall picture of the ability of different viruses to modulate Treg activation and the immune system in early life.

In conclusion, our results reveal that immunoregulatory mechanisms are up-regulated after rhinovirus infections, while enterovirus infections are associated with activation of proinflammatory pathways and decreased immune regulation. Further studies are needed to evaluate the significance of these phenomena in the development of virus-induced immune pathologies and possible role in the development of autoimmune and/or allergic diseases. The results suggest that early virus infections may affect the function of immunoregulatory cells and that this effect may last long after the infection. To our best knowledge, this is the first study showing such an effect during longitudinal follow up of young children.

## Data Availability Statement

The raw data supporting the conclusions of this article will be made available by the authors, without undue reservation.

## Ethics Statement

The studies involving human participants were reviewed and approved by HUS, Ethics committee II, Dnr: 228/13/03/03/2008. Written informed consent to participate in this study was provided by the participants’ legal guardian/next of kin.

## DIABIMMUNE Study Group

The following Authors, who are listed in alphabetical order, contributed to the work of the DIABIMMUNE Study Group;

**Mikael Knip**, **Taina Härkönen**, **Samppa Ryhänen and Heli Siljander**, Children’s Hospital, University of Helsinki and Helsinki University Hospital, Helsinki, Finland; **Katriina Koski**, **Matti Koski**, **Janne Nieminen**, **Terhi Ruohtula**, and **Outi Vaarala**, Clinicum, University of Helsinki, Helsinki, Finland; **Onni Niemelä**, Department of Laboratory Medicine and Medical Research Unit, Seinäjoki Central Hospital and University of Tampere, Seinäjoki, Finland; **Anu-Maaria Hämäläinen**, Jorvi Hospital, Helsinki University Hospital, Espoo, Finland; **Anne Ormisson**, Children’s Clinic, Tartu University Hospital, Tartu, Estonia; **Aleksandr Peet and Vallo Tillmann**, Department of Pediatrics, Tartu University Hospital, Tartu, Estonia; **Valentina Ulich**, **Elena Kuzmicheva and Sergei Mokurov**, Ministry of Health and Social Development, Karelian Republic of the Russian Federation, Petrozavodsk, Russia; **Svetlana Markova and Svetlana Pylova**, Children’s Republic Hospital, Karelian Republic of the Russian Federation, Petrozavodsk, Russia; **Marina Isakova and Elena Shakurova**, Perinatal Center, Karelian Republic of the Russian Federation, Petrozavodsk, Russia; **Vladimir Petrov**, Maternity Hospital No. 1, Petrozavodsk, Russia; **Natalya V. Dorshakova**, **Tatyana Karapetyan and Tatyana Varlamova**, Petrozavodsk State University, Petrozavodsk, Russia; **Jorma Ilonen and Minna Kiviniemi**, Immunogenetics Laboratory, University of Turku and Turku University Hospital, Turku, Finland; **Kristi Alnek, Helis Janson and Raivo Uibo**, Department of Immunology, University of Tartu, Tartu, Estonia; **Tiit Salum**, OÜ Immunotron, Tartu, Estonia; **Erika von Mutius and Juliane Weber**, Children’s Hospital, Ludwig Maximilians University, Munich, Germany; **Helena Ahlfors**, **Henna Kallionpää**, **Essi Laajala, Riitta Lahesmaa**, **Harri Lähdesmäki and Robert Moulder**, Turku Centre of Biotechnology, University of Turku and Åbo Akademi University, Turku, Finland; **Heikki Hyöty**, **Hanna Honkanen**, **Anita Kondrashova and Sami Oikarinen**, Department of Virology, University of Tampere, Tampere, Finland, **Hermie J. M. Harmsen**, **Marcus C. De Goffau and Gjalt Welling**, University Medical Center Groningen, Groningen, the Netherlands; **Kirsi Alahuhta and Suvi M. Virtanen**, Department for Welfare and Health Promotion, National Institute for Health and Welfare, Helsinki, Finland.

## Author Contributions

TR, AK, HH, JN, OV, and MK planned, performed, and analyzed experiments. TR, AK, and SO performed data analysis, and JL performed statistical analyzes. JN and SO were involved in data discussion and supervision of study design experiments. A-MH, AP, VT, and ON, collected and provided patient and/or control material and clinical characterization. JN, AK, TR, and SO planned experiments. JI provided scientific and experimental input. MK was involved in study design, data discussion, and supervision of experiments. OV and HH designed experiments analyzed data and supervised the study. OV, TR, and AK wrote the manuscript. All authors contributed to the article and approved the submitted version.

## Funding

Our study was supported by the Academy of Finland Centre of Excellence in Molecular Systems Immunology and Physiology Research (250114), Novo Nordisk Foundation (grant 34080), Sigrid Juselius Foundation, Diabetes Research Foundation in Finland, Tampere Tuberculosis Foundation, and Academy of Finland (grant 288671).

## Conflict of Interest

The authors declare that the research was conducted in the absence of any commercial or financial relationships that could be construed as a potential conflict of interest.
