# Supplementary material for: Immunomodulatory Effects of Rhinovirus and Enterovirus Infections During the First Year of Life
Source: Front Immunol. 2021 Feb 11;11:567046. doi: 10.3389/fimmu.2020.567046 (PMC7905218; doi:10.3389/fimmu.2020.567046)
Supplement: Supplementary file 1 [file DataSheet_1.pdf]

Supplementary Material

1 Supplementary Figures

Supplementary Figure 1. Gating protocols are shown for the different regulatory T cell populations.

Supplementary Figure 2. Age-related changes in circulating T-cell phenotype related cytokines.

2 Supplementary Tables

Supplementary Table 1. The primers and probes and the concentration of the oligonucleotides in the qPCR reactions

Supplementary Table 2. Product information on the reagents used in the study.

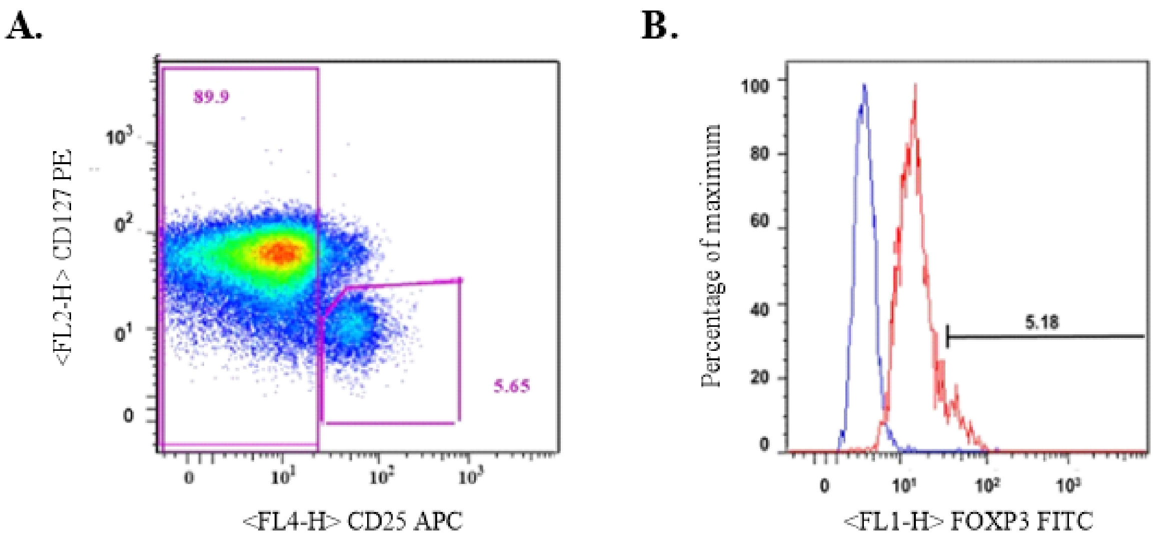

Supplementary Figure 1. Gating protocols are shown for the different regulatory T cell populations. (A) Regulatory T cells (Treg) in CD4+ are defined as CD25+CD127-/lo. Shown as the smaller gate at the lower right. (B) A histogram of FOXP3 fluorescence intensity demonstrates a population of Treg cells with a high expression of FOXP3 (TregFOXP3high) in the FOXP3 positive Treg cell population. The gate is shown as a horizontal line in the graph and the negative isotype control as a histogram on the left.

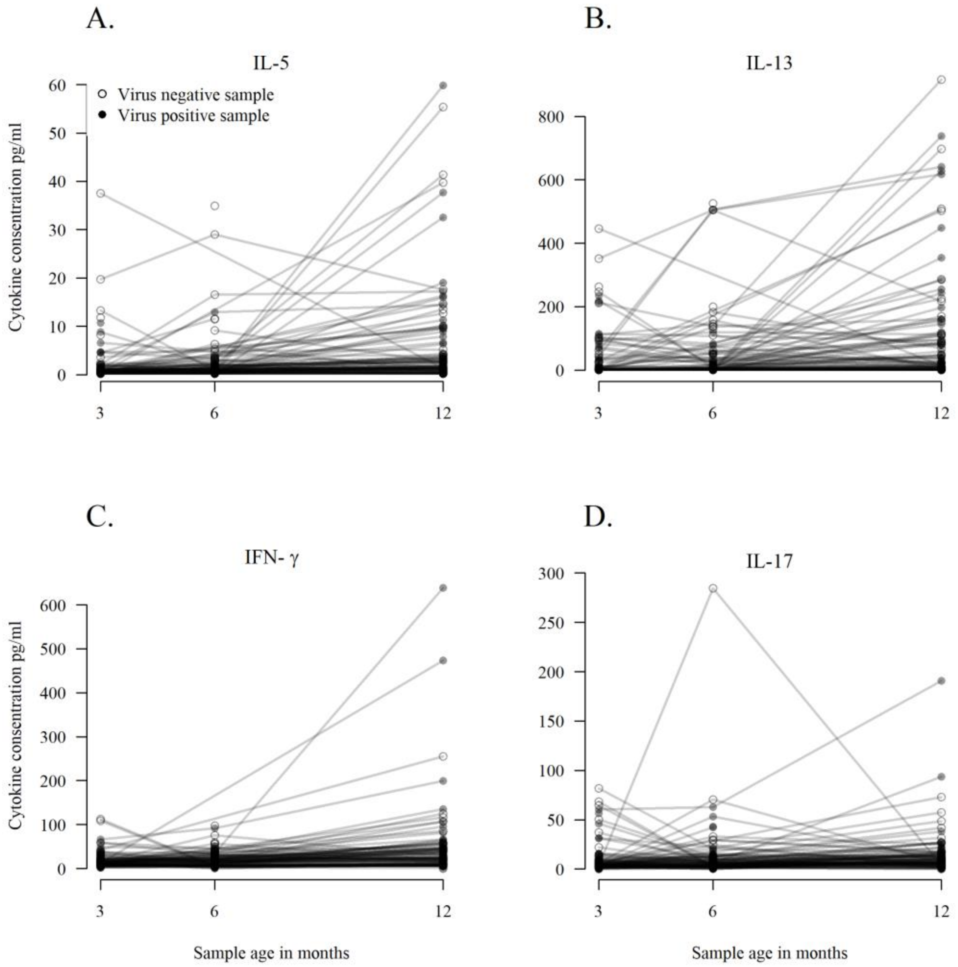

Supplementary Figure 2. Circulating T-cell phenotype related cytokines increase with age. The cytokine levels are shown for each infant. (A) IL-5, (B) IL13, (C) IFNγ, and (D) IL17A. Black circles and lines represent virus positive samples, and open circles represent virus negative samples.

Supplementary Table 1. The primers and probes and the concentration of the oligonucleotides in the qPCR reactions

Virus	Primer name	Sequence	Concentration (µM)
Enterovirus	fwd 636	CGGCCCCTGAATGCGGCTAA	900
	rev 4-	GAAACACGGACACCCAAAGTA	900
	Q-PCREVI	FAM-TCTGTGGCGGAACCGACTA-TAMRA	300
	Q-PCREVII	FAM-TCTGCAGCGGAACCGACTA-TAMRA	300
Rhinovirus	Rhinofwd	CP+AGCC+TGCGTGGC	900
	RhinoRev	GAAACACGGACACCCAAAGTA	900
	RhinoProbe	VIC-TCCTCCGGCCCCTGAATGYGGC –TAMRA	300
Norovirus	NoroG2 fwdQ	CARGARBCNATGTTYAGRTGGATGAG	900
	NoroG2 revQ	TCGACGCCATCTTCATTCACA	300
	NoroG1 revQ	CTTAGACGCCATCATCATTYAC	900
	NoroG1 fwdQ	CGYTGGATGCGNTTYCATGA	900
	NoroG2proQ	FAM-TGGGAGGGCGATCGCAATCT-TAMRA	300
	NoroG1proQ	VIC-AGATYGCGATCYCCTGTCCA- TAMRA	250
Rotavirus	VP2-F1	TCTGCAGACAGTTGAACCTATTAA	900
	VP2-F2	CAGACACGGTTGAACCCATTAA	900
	VP2-F3	TCGGCTGATACAGTAGAACCTATAAATG	900
	VP2-F4	TGTCAGCTGATACAGTAGAACCTATAAATG	900
	VP2-F5	TCAGCTGACACAGTAGAACCTATA AATG	900
	VP2-R1	GTTGGCGTTTACAGTTCGTTCAT	50
	VP2-R2	GTTGGCGTCTACAATTCGTTCAT	50
	RotaVp2-P	FAM-ATG CGC ATR TTR TCA AAH GCA A-MGB-NFQ	200
Parechovirus	ParE AN345	GTAACASWWGCCTCTGGGSCCAAAAG	300
	ParE AN344	GGCCCCWGRTCAGATCCAYAGT	300
	ParE AN257	FAM-CCTRYGGGTACCTYCWGGGCATCCTTC-TAMRA	200

Supplementary Table 2. Product information on the reagents used in the study.

Product	clone	Product code	Supplier
perCP-anti-human-CD4	SK3	345770	BD Biosciences, Franklin Lakes, NJ, USA
APC-anti-human-CD25	M-A251	555434	BD Biosciences, Franklin Lakes, NJ, USA
PE-anti-human-CD127	HIL-7R-M21	557938	BD Biosciences, Franklin Lakes, NJ, USA
Alexa-488-anti-human-FOXP3	206D	320111	BioLegend, San Diego, CA, USA
Alexa488-Mouse-IgG1-isotype		400133	BioLegend, San Diego, CA, USA
SPHERO™ Easy Calibration Fluorescent Particles, FITC		ECFP-F1-5K	Spherotech, Libertyville, IL, USA
SPHERO™ Easy Calibration Fluorescent Particles, PE		ECFP-F2-5K	Spherotech, Libertyville, IL, USA
SPHERO™ Easy Calibration Fluorescent Particles, APC		ACP30-5K	Spherotech, Libertyville, IL, USA
GE Healthcare's Ficoll-Paque density gradient		17-1440-03	Amersham Biosciences, Uppsala, Sweden
FACS Lysing Solution		349202	BD Biosciences, San Jose, CA
FACSCalibur™			BD Bioscience2
FlowJo™ software			Tree Star, Ashland, OR, USA
MILLIPLEX MAP Human Cytokine/Chemokine Magnetic bead 38-plex Panel		HCYTOMAG-60K	Millipore, Billerica, MA, USA
Bio-Rad Bio-Plex 200 System™			Bio-Rad Laboratories, Hercules, CA, USA
Bio-Plex Manager 5.0 program			Millipore, Billerica, MA, USA
Graph Pad Prism 5 software			La Jolla, CA, USA
MagNaPure extraction robot			Roche Diagnostics, Mannheim, Germany
Total Nucleic Acid extraction kit			Roche Diagnostics, Mannheim, Germany
QuantiTect Probe kit			Qiagen, Germany
